# Evaluation of the NovaLisa™ *Leishmania Infantum* IgG ELISA in A Reference Diagnostic Laboratory in A Non-Endemic Country

**DOI:** 10.3390/antib8010020

**Published:** 2019-02-27

**Authors:** Christen Rune Stensvold, Amalie Vang Høst, Salem Belkessa, Henrik Vedel Nielsen

**Affiliations:** 1 Department of Bacteria, Parasites & Fungi, Statens Serum Institut, Artillerivej 5, DK–2300 Copenhagen S, Denmark; amalievhoest@gmail.com (A.V.H.); salembelkessa@yahoo.com (S.B.); hvn@ssi.dk (H.V.N.); 2 Department of Biochemistry and Microbiology, Faculty of Biological and Agronomic Sciences, Mouloud Mammeri University of Tizi Ouzou, 15000 Tizi Ouzou, Algeria; 3 Department of Natural and Life Sciences, Faculty of Exact Sciences and Natural and Life Sciences, Mohamed Khider University of Biskra, 07000 Biskra, Algeria

**Keywords:** parasite, clinical microbiology, vector-borne disease, leishmaniasis, diagnosis, diagnostic methods, molecular epidemiology

## Abstract

Anti-*Leishmania* antibodies may be detectable in patients with leishmaniasis. Here, we compared a commercial enzyme-linked immunosorbent assay (ELISA) for the detection of anti-*Leishmania* antibodies, with an immunofluorescence antibody test (IFAT) that is no longer commercially available. Eighty-six serum samples from 73 patients were tested. The results obtained by the NovaLisa™ *Leishmania infantum* IgG ELISA, interpreted according to the instructions of the manufacturer, but with a modified cut-off for borderline positive values, were compared with the IFAT results that were already available. Moreover, *Leishmania* Western blot IgG results were available for 43 of the samples. The overall concordance of ELISA and IFAT was 67%. The ELISA and IFAT tests scored as 24% and 15% of the samples being positive, respectively, while 13% and 33% scored as borderline-positive, respectively. Using a Western blot (WB) as the reference, the sensitivities and specificities for the positive plus borderline-positive samples combined was 95.5% (95% confidence interval (CI), 77.2–99.9%) and 81.0% (95% CI, 58.1–94.6%) for ELISA, and 95.5% (95% CI, 77.2–99.9%) and 42.9% (95% CI, 21.8–66.0%) for IFAT, respectively. Overall, the ELISA proved to be a cost-effective alternative to the IFAT, due to its higher accuracy and specificity, and with a consequently lower number of confirmatory WB tests being required. Lastly, we also present data on the associations between seroconversion and the type of leishmaniasis.

## 1. Introduction

Leishmaniasis occurs endemically in more than 90 countries [1]. The main clinical manifestations include visceral and cutaneous leishmaniasis. In 2015, more than 90% of global cases were reported by only seven countries (Brazil, Ethiopia, Kenya, Somalia, South Sudan, and Sudan) [2]. Nevertheless, climate change, changes in demographics (e.g., a rise in immigrants from highly endemic countries), increased travel to endemic regions, and improved diagnostic methods and algorithms are all factors resulting in an increased awareness of leishmaniasis in countries where the number of cases was previously very low, such as Denmark [3,4].

Laboratory diagnosis of leishmaniasis relies mainly on direct (microscopy or DNA-based detection) and indirect (serology) detection. Until recently, a commercially available serological test, the immunofluorescence antibody test (IFAT, Leishmania-spot IF; bioMérieux, Marcy l’Etoile, France) was available for the detection of anti-*Leishmania* antibodies; this test, however, is no longer available for purchase. In the present study, we set out to evaluate a commercial enzyme-linked immunosorbent assay (ELISA) for the detection of anti-*Leishmania* antibodies, using the IFAT and Western blot as reference methods. A secondary goal aimed to identify the associations between antibody responses detectable by the IFAT (seroconversion) and the infecting species, as confirmed by polymerase chain reaction (PCR) and sequencing in those patients, for whom results from both serological and DNA-based tests were available.

## 2. Materials and Methods

Between January 2002 and August 2017 (this will be referred to as ‘the study period’), 1,726 samples from 1466 patients were tested for *Leishmania* at the Laboratory of Parasitology, Statens Serum Institut, Copenhagen, comprising 313 blood/biopsy samples from 262 patients tested by real-time PCR, and 1413 serum samples from 1320 patients tested for anti-*Leishmania* antibodies by an immunofluorescence antibody test (IFAT). Samples available for PCR included genomic DNAs extracted from skin biopsies, bone marrow, ethylenediaminetetraacetic acid (EDTA) blood, and other patient materials (see below) using either the DNeasy Blood & Tissue Kit or a QIAcube (QIAGEN, Hilden, Germany).

### 2.1. PCR and Sequencing

Our real-time PCR used the primers LEIS.U1 (5′-AAGTGCTTTCCCATCGCAACT-3′) and LEIS.L1 (5′-GACGCACTAAACCCCTCCAA-3′), and the probe LEIS.P1 (5′-CGGTTCGGTGTGTGGCGCC-3′) [5], targeting nuclear small subunit ribosomal DNA. For species identification, the ITS1 region was amplified and sequenced, using the primers LITSR (5′CTGGATCATTTTCCGATG-3′) and L5∙8S (5′-TGATACCACTTATCGCACTT-3′) [6].

### 2.2. Routine Serological Testing

In the study period, routine diagnostic testing for anti-*Leishmania* antibodies relied on a commercially available indirect immunofluorescent antibody test (IFAT (*Leishmania*-spot IF; bioMérieux, Marcy l’Etoile, France)). The test was carried out according to the recommendations of the manufacturer. Sera were considered positive for anti-*Leishmania* antibodies when the IgG titer was equal to or above 1:160. Titers of 1:40 and 1:80 were considered ‘borderline-positive’.

### 2.3. Evaluation of the Leishmania Infantum IgG ELISA

Patient samples that had tested positive or borderline-positive according to IFAT, were collected for the study. Furthermore, the latest available patient samples that had tested negative by the IFAT method were also collected for the study. This led to the inclusion of 86 serum samples from 73 patients, for the evaluation of the *Leishmania infantum* IgG ELISA test. Hence, the IFAT results already available from previous routine diagnostic testing (see 2.2.) were used for comparison (Table S1). 

Moreover, for 26/86 samples, a *Leishmania* PCR result was also available (i.e., a PCR had been performed on DNA extracted from a tissue biopsy, or on an EDTA blood/bone marrow sample from the patient submitting the blood sample (serum) for antibody testing).

The ELISA test was performed in duplicates, and the average of the two test values produced for each sample was used as the final test result. A *t*-test was used to identify the extent of reproducibility, and test accuracies (indicating the overall probability that a patient will be correctly classified) were calculated and compared (the template used is available online at https://www.socscistatistics.com/tests/ttestdependent/Default2.aspx).

ELISA results were interpreted according to the instructions of the manufacturer. However, based on the observations made during the implementation of the ELISA assay in the laboratory, it was decided that the limit of borderline-positivity should be lowered, and so a range of 7–11 NOVATEC units were used to indicate ‘borderline-positive’ values instead of 9–11 recommended by the manufacturer. 

Western blot (WB) results (scored as ‘positive’ or ‘negative’) were available for 43 of the samples, and these were obtained using LEISHMANIA Western blot IgG (LDBIO Diagnostics, Lyon, France).

Dates for the PCR, IFAT, and ELISA tests were available.

### 2.4. Ethical Considerations

All of the samples were samples that were previously submitted for *Leishmania* serology testing, and none of the samples used were used for purposes other than that originally intended. No personal data (i.e., data on, for example, age, gender, names, ethnicity, etc.) pertaining to the tested sera were used in the analyses, or published in this article.

## 3. Results

### 3.1. Anti-*Leishmania* Antibody Test Results (IFAT) and *Leishmania* Species-Specific PCR Results According to Sample Localization

Samples tested by PCR comprised biopsies from the skin (62%), bone marrow/EDTA blood (22%), mucous membranes (4%), and other materials (12%). A total of 70/313 (22.4%) samples tested by PCR were positive, corresponding to 55 patients. For 116/262 patients tested by PCR, IFAT results were available. For 26 of the 55 PCR-positive patients, IFAT results were available, as follows: Six patients (23%) were seropositive, six (23%) had borderline-positive titers, and the remaining 14 (54%) were classified as being seronegative (Table 1). For 8/12 (67%) IFAT-positive and PCR-positive patients (Table 1), DNA from ‘deep biopsies’ (bone marrow/EDTA blood/biopsies from internal organs) were submitted to PCR. The six patients who were seropositive were all infected with *L. donovani*/*L. infantum.* With regard to the six patients with IFAT borderline-positive titers, two were infected by *L. donovani*/*L. infantum*, and four with *L. tropica*. Anti-*Leishmania* antibodies were not detected in patients with *Leishmania* of the Viannia group, nor in patients with *L. major* and *L. mexicana*.

### 3.2. Comparison of ELISA with IFAT and WB

Results obtained by ELISA and IFAT for the 86 serum samples tested by both methods are displayed in Table 2.

In 67% of the cases, the samples were scored as either positive, borderline-positive, or negative by both tests. In the remaining 23% of the cases, the two tests were not in agreement. The ELISA and IFAT tests scored 21 (24%) and 13 (15%) of the samples as positive, respectively.

Of the 21 ELISA-positive samples, nine were IFAT-positive, 11 fell into the IFAT borderline-positive range, and only one was IFAT-negative; the sample that was ELISA-positive and IFAT-negative was positive according to WB. Moreover, out of the 21 ELISA-positive samples, 18 were tested by WB, of which 16 were positive.

Of the 13 IFAT-positive samples, nine samples were tested by WB, eight of which were positive.

None of the 54 ELISA-negative samples were IFAT-positive; however, 11 of these samples were categorized as IFAT borderline-positive. Of these 11 samples, WB results were available for 10, and WB was positive only in one case. The ELISA value for this serum sample was 4.74, and hence it was nowhere near the cut-off for being borderline-positive; this patient had no sample analyzed by PCR.

Considering only the 43 samples that had been tested by WB, and merging the results for ‘positive’ and ‘borderline-positive’ for ELISA and IFAT (Table 2), the calculated values for sensitivity, specificity, positive predictive value, and negative predictive value for the ELISA were 95.5% (95% CI, 77.2–99.9%), 81.0% (95% CI, 58.1–94.6%), 84.0% (95% CI, 68.4–92.7%), and 94.4% (95% CI, 71.2–99.2%), respectively. The corresponding values for the IFAT were 95.5% (95% CI, 77.2–99.9%), 42.9% (95% CI, 21.8–66.0%), 63.6% (95% CI, 54.4–71.9%), and 90.0% (95% CI, 55.5–98.5%), respectively. The test accuracies for the ELISA and the IFAT were 88.4% (95% CI, 74.9–96.1%) and 69.8% (95% CI, 53.9–82.8%), respectively.

Looking at the 26 samples for which PCR results were available (Table 3), it appeared that the ELISA titers were scored as being positive (*n* = 8) or borderline-positive (*n* = 2) in 10/11 samples stemming from patients that were tested as PCR-positive for *L. donovani/L. infantum*. Meanwhile, the IFAT scored six samples as positive, and four samples as ‘borderline-positive’. The remaining serum sample from a *L. donovani/L. infantum*-positive patient that scored negative according to the ELISA, was also negative by IFAT. This patient had been sampled twice over a period of five months; both times, a biopsy from a cheek ulcer had been submitted for PCR testing.

With regard to the five serum samples for which PCR tests and the sequencing of samples from the respective patients revealed *L. tropica*, two samples were positive by ELISA, one was borderline-positive, and two were negative. For the IFAT, the corresponding results were zero positive samples, four borderline-positive, and one negative (Table 3; for more information, please refer to Supplementary Table S1).

The Average ELISA values for positive samples ranged from 11.25 to 28.89, with a mean and median of 19.77 and 21.99 (interquartile range (IQR), 14.63–24.29), respectively.

Two of the 21 ELISA-positive samples were WB-negative. These two samples were from the same patient.

## 4. Discussion

The data obtained during the evaluation of the *Leishmania infantum* IgG ELISA test suggest that this assay is an attractive alternative to the IFAT test, against which is was tested. First and foremost, the ELISA was characterized by having a higher test accuracy than that of the IFAT, although not significantly so. In almost 9 out of 10 cases, a given patient is correctly classified when using the ELISA alone, whereas for the IFAT, this ratio is only 7/10. Moreover, the number of definitive positives that are identified by the ELISA are much higher (*n* = 21) than for the IFAT (*n* = 13). Although this difference is non-significant at the 5% level, this finding means that the number of WB tests that are required is far lower for the ELISA than for the IFAT, if WB is used only in cases of borderline positivity.

Secondly, the ELISA appears to be reproducible, and it does not entail the specific level of expertise that is required for calling test results when using immunofluorescence tests.

Although potentially unsurprisingly, a positive serology was associated primarily with infections by species causing visceral leishmaniasis, rather than from species causing cutaneous leishmaniasis. However, this picture does not appear to be clearly ‘black and white’. One patient with PCR-confirmed *Leishmania donovani*/*infantum* was tested as sero-negative (confirmed by both ELISA and IFAT), while some patients with cutaneous leishmaniasis were seropositive. This means that serology testing alone is not always sufficient to rule out infections due to *Leishmania donovani*/*infantum*.

Since we only used the ITS1 region for species identification, we were not able to tell apart *L. donovani* from *L. infantum*. It is possible that the serological responses towards each of these two species could differ.

The number of studies that have used the NovaLisa Leishmania Infantum IgG ELISA assay is very limited. It was recently used by Šiško-Kraljević and colleagues in a study surveying the seroprevalence of leishmaniasis among asymptomatic individuals in Croatia; however, no confirmatory tests were used. The study scored only samples as being negative or positive, and so no ‘borderline-positive’ scores were used [7].

## 5. Conclusions

For screening, the NovaLisa™ *Leishmania infantum* IgG ELISA is useful as an alternative to the IFAT with a modified range for borderline-positivity, as indicated in the present study, and combined with WB for the confirmation of borderline-positive samples.

## Figures and Tables

**Table 1:** Antibody test (IFAT) results according to *Leishmania* species, as confirmed by polymerase chain reaction (PCR) and the sample material.

Leishmania Species	PCR-Positive Patients	IFAT-Tested Patients	IFAT-Positive (Borderline-Positive)	Sample Material
*L. braziliensis/* *L. guyanensis/* *L. panamensis*	7	2	0	Skin/wound
*L. donovani/* *L. infantum*	17	12	6 (2)	Bone marrow, ethylenediaminetetraacetic acid (EDTA) blood, skin/wound
*L. major*	6	2	0	Skin/wound
*L. mexicana*	1	0	0	Skin/wound
*L. tropica*	22	8	(4)	Skin/wound, brain, uvula, epiglottis
Species determination not possible	2	2	0	Skin/wound
Total	55	26	12	Bone marrow, EDTA blood, skin/wound, brain, uvula, epiglottis

**Table 2:** Results from enzyme-linked immunosorbent assay (ELISA) and immunofluorescence antibody test (IFAT) screening of the 86 serum samples tested in the study. The numbers in parentheses indicate the total number of samples tested by Western blot (WB)/number of samples listed as positive by WB.

	**ELISA-Positive**	ELISA-Borderline- Positive *	**ELISA-Negative**	**TOTAL**
**IFAT-positive**	9 (6/6)	4 (3/2)	0	13 (9/8)
**IFAT-borderline positive**	11 (11/9)	6 (3/3)	11 (10/1)	28 (24/13)
**IFAT-negative**	1 (1/1)	1 (1/0)	43 (8/0)	45 (10/1)
**TOTAL**	21 (18/16)	11 (7/5)	54 (18/1)	86 (43/22)
	**ELISA-Positive or Borderline-Positive**		**ELISA-Negative**	**TOTAL**
**IFAT-positive or -borderline positive**	30 (23/20)		11 (10/1)	41 (33/21)
**IFAT-negative**	2 (2/1)		43 (8/0)	45 (10/1)
**TOTAL**	32 (25/21)		54 (18/1)	86 (43/22)

* ELISA results were interpreted according to the instructions of the manufacturer, apart from the fact that the 7–11 NOVATEC unit range was interpreted as indicating weak seropositivity, instead of the 9–11 NOVATEC unit range recommended by the manufacturer.

**Table 3:** For 26/86 serum samples tested by both ELISA and IFAT, a PCR result was available (please see text for details).

	*L. donovani/L. infantum* PCR-Positive	*L. tropica* PCR-positive	PCR-Positive (Unknown Species)	PCR-Negative
IFAT (positive/borderline-positive/negative)	6/4/1	0/4/1	0/1/1	1/3/4
ELISA (positive/borderline-positive/negative)	8/2/1	2/1/2	0/1/1	3/0/5

The means and medians of the differences in results between the first and second ELISA run was 0.61 and 0.33 (interquartile range (IQR): 0.16–0.65), respectively. With regard to the reproducibility of the ELISA, an analysis of duplicate values gave a mean difference of 0.12 between the paired values, with a *t* value of 1.031339 (*p* = 0.305), indicating that the ELISA test results were reproducible.

## Supplementary Materials

The following are available online at https://www.mdpi.com/2073-4468/8/1/20/s1, Table S1: Raw data from the comparison of the ELISA and IFAT tests.
